# Recent Themes in Social Networking Service Research

**DOI:** 10.1371/journal.pone.0170293

**Published:** 2017-01-20

**Authors:** John S. Liu, Mei Hsiu-Ching Ho, Louis Y. Y. Lu

**Affiliations:** 1 Graduate Institute of Technology Management, National Taiwan University of Science and Technology, Taipei, Taiwan; 2 College of Management, Yuan Ze University, Chung-Li, Taoyuan, Taiwan; Nanyang Technological University, SINGAPORE

## Abstract

The body of literature addressing the phenomenon related to social networking services (SNSs) has grown rather fast recently. Through a systematic and quantitative approach, this study identifies the recent SNS research themes, which are the issues discussed by a coherent and growing subset of this literature. A set of academic articles retrieved from the Web of Science database is used as the basis for uncovering the recent themes. We begin the analysis by constructing a citation network which is further separated into groups after applying a widely used clustering method. The resulting clusters all consist of articles coherent in citation relationships. This study suggests eight fast growing recent themes. They span widely encompassing politics, romantic relationships, public relations, journalism, and health. Among them, four focus their issues largely on Twitter, three on Facebook, and one generally on both. While discussions on traditional issues in SNSs such as personality, motivations, self-disclosure, narcissism, etc. continue to lead the pack, the proliferation of the highlighted recent themes in the near future is very likely to happen.

## 1. Introduction

Social networking services (SNSs), denoting web-based services that provide users with social interaction and microblogging functions, have suddenly become a large part of people’s life in recent years. One research report [1] estimates that 72% of American online adults in 2015 are Facebook users, while the same estimations for Pinterest, Instagram, LinkedIn, and Twitter are 31%, 28%, 25%, and 23%, respectively—all of which saw significant growth. Such an overwhelming usage of SNSs has led scholars to study the cause, nature, and impact of the phenomenon and attempt to answer questions like who these SNS users are, why and how they use the services, and what the influences of SNSs have on their users and the society as a whole. In less than 10 years, the related academic articles on SNSs have gradually formed a large body of literature worth a detailed examination.

There have been many articles examining this body of SNS literature. Among them, some offer a general overview [2, 3]; some focus on a particular site such as Facebook [4, 5] or Twitter [6]; while others take on specific topics—for example, psychological determinants [7], addiction [8], privacy [9], educational environments [10, 11], health [12], etc. Virtually all these review-type articles organize their discussions on research themes at the authors’ discretion. Table 1 summarizes some of these articles along with the themes they highlight and discuss. From the table, one can see that themes like user characteristics, motivations, disclosure, and privacy are mentioned in many of these review articles, suggesting that these themes are ‘existing themes’, i.e. they have been discussed extensively in earlier articles. The purpose of this article is to highlight the recent themes in SNS research. Recent themes, in contrast to existing themes, are those topics that just bring to the attention of scholars. While they are not extensively studied, some recently published works have started to accumulate a small body of coherent literature.

**Table 1:** Topics of existing research on SNSs.

Themes	*Boyd and Ellison [2]*	Berger et al. [3]	Wilson et al. [4]	Cheong and Lee [6]	Kuss and Griffiths [8]	Caers et al. [5]	Hugl [9]	Manca and Ranieri [10]	Aydin [11]	Blachnio et al. [7]	Finfgeld-Connett [12]
Usage/User characteristics			✓	✓	✓	✓			✓	✓	
User behavior		✓							✓		
Motivations	✓		✓		✓	✓			✓	✓	
Disclosure (what, why, how, and effects)			✓			✓	✓			✓	
Effects of SNSs (self-efficacy)											
Negative correlates					✓				✓		
Friendship performance	✓										
Networks and network structure	✓										
Addiction					✓						
Bridging online and offline social networks	✓										
Privacy	✓	✓	✓				✓				
Characteristics of SNSs		✓									
Educational environment									✓		
SNS in organizations and society		✓				✓					
Role in social interaction			✓								
Message analysis				✓							
Health											✓

Most of the review-type articles that highlight recent themes are based on subjective observations supported by the authors’ expertise and/or through qualitative analysis of research contents. In this study we uncover recent themes through a systematic and quantitative method. More specifically, we apply a citation-based bibliometric method to answer our research question: What are the recent themes in SNS research? We construct a citation network and apply a widely used network clustering method [13, 14] to divide the citation network into subnetworks, each of which has its nodes tightly knitted within but loosely connected to others outside. Articles within each subnetwork thus very likely consist of studies that elaborate on a common research theme.

This study can be categorized as a review-type article, but it deviates from previous similar studies in three aspects. First, it focuses on recent research themes, which in our definition are the issues discussed by a small set of coherent and growing studies in the literature. Second, it is a comprehensive review and covers research on all social networking services and on all subjects in social science, including psychology, business, management, etc. Third, it is a large-scale citation-based study that takes over two thousand academic articles into consideration and separates them into groups according to their citation relationship.

In the following sections, we first introduce the methodology, mainly the edge-betweenness network clustering method. It is followed by descriptions on how data is collected. The next section presents clustering results and elaborates on each recent theme. We then discuss potential oversights of our study. The concluding section suggests future research directions.

## 2. Methods

Referencing previous works is a standard practice in publishing the results of scientific research. This practice allows one to construct from a collection of target articles a citation network in which nodes are articles and links are the referencing relationships among the articles. Citation links in a citation network are usually assigned a direction pointing from the earlier to the later articles, indicating that knowledge is diffused from the earlier articles to the newer articles.

When scholars publish an article on a theme they are most likely aware of studies with similar ideas and reference these studies in their publication. After a period of time, articles that address similar themes will connect to each other through citation links and gradually form a tightly knitted web. Within each of these webs, articles reference more works with a similar theme than those with remotely related themes. In network analysis language, each of these webs is a subnetwork and has heavy links among nodes within yet little connection to nodes outside. We therefore are able to detect recent themes from the citation network of a scientific field by identifying subnetworks in a citation network.

It should be noted, however, that not all subnetworks embed recent themes. A relatively large-size subnetwork probably embeds not a current theme, but rather an existing theme. Moreover, it may be too early to call for a recent theme from a subnetwork that is relatively small. Another condition for identifying recent themes is article growth. As an active and appealing subfield, the subnetwork that embeds recent themes has to be growing in time, compared to subnetworks that have their sizes decrease in time embedding themes of lessen attention. Thus, we look for two signs for recent research themes: a coherent subnetwork of proper size and growth in the number of publications.

The citation network is inherently directed and acyclic, implying that each connection is only meaningful in one direction (directed) and that no one chain of connections loops back to where it starts after following the defined direction (acyclic). To separate a citation network into subnetworks, the most common approach is naively transforming the network into an undirected network by simply ignoring directionality [15]. The advantage of taking this approach is that many well-recognized methods exist to cluster an undirected network. The downside, however, is that the information of endorsement from one node to another is neglected. In recent years, the literature has proposed many new methods to cluster a directed network [15–25], including naïve network transformation, transformations maintaining directionality, extending an existing method for a directed network, and other innovative new approaches [15]; among them, only a few demonstrate their methods using citation networks [20, 23]. Moreover, only one of them specifically addresses the directed acyclic network [25], by proposing ‘modularity’ (quality measure for clustering results) for such a network and applying the method to two citation networks among others. This study finds that the resulting modularity values are similar, whether directionality is taken into account or not, and that the resulting subnetworks are also fairly similar when compared against those reported elsewhere. In summary, the methods to cluster directed acyclic networks are still undergoing a fast development, and “clustering problems in directed networks is more challenging compared to the undirected version” [15]. We therefore adopt a rather conservative approach and treat the citation network as an undirected network.

The method this study employed to cluster the target citation network is the widely used edge-betweenness clustering [13, 14, 26, 27], which in association with the network modularity [28] concept identifies those subnetworks that are coherent in their citation relationships. The method finds subnetworks by gradually breaking up a network through cutting the network links that have high edge-betweenness, or in other words, removing links that play a significant bridging role. Unlike traditional clustering methods such as K-means that require a pre-specified number of clusters, edge-betweenness clustering determines the number of clusters automatically through modularity [28], which is a quality measure for network division. A network with high modularity is dense in connections between the nodes within subnetworks, but sparse in connections between nodes in different subnetworks. Among many possible divisions uncovered in the link-removing process, the optimal division for a network is the one that has the largest value of network modularity. The modularity concept relieves the method from specifying the number of subnetworks in advance. We adopt the igraph [29] implementation of the edge-betweenness clustering algorithm in the Microsoft Visual C++ development environment.

## 3. Data

This study obtained articles and citation data from the Web of Science (WOS) service provided by Thomson Reuters. Data collection was conducted in two steps. It began with a search in WOS using a query string consisting of SNS related terms. These terms are designed by referencing search terms used by several review articles on SNS [3, 30–32], Phrases like “online social network*”, “Internet social network*”, "social network* site*", "social network* website*", "social network* web site*”, “social network* service*”, “social network* web*”, and specific services such as Facebook, Twitter, Myspace, Instagram, LinkedIn, etc. are included in the query string. Noting that the wildcard character ‘*’ allows variations of a specified term. Articles that have their title meet one of the conditions specified in the query string were retrieved from the WOS Social Science Citation Index (SSCI) database. We retrieve data from SSCI, because we are interested in the development of the social science literature. This step returned 2,445 articles on January 30, 2016.

The second step removed irrelevant articles, which are articles that receive no citation from and do not cite articles within the dataset obtained in the first step. These articles are regarded as noises, because they are not associated with other articles from the citation point of view. The remaining 2,291 articles become the target of this study.

## 4. Recent Research Themes

**Fig 1:**
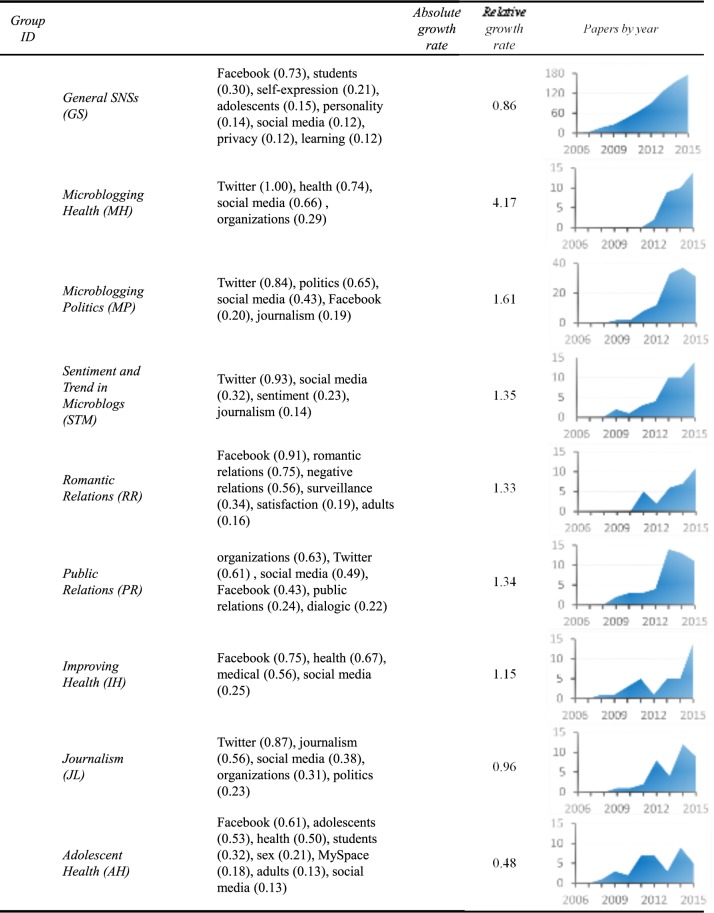
Themes in SNS research. Numbers in the parentheses are the average keyword count per article in the titles and abstracts. Only those keywords with an average appearance count greater than 0.12 are listed.

Edge-betweenness clustering divides the citation network of SNS literature into many subnetworks, each consisting of a group of articles that cite each other more than those outside the group. The top nine largest groups are 739, 129, 51, 44, 39, 38, 36, 35, and 32 in size, while the sizes of the remaining groups are no greater than 28. The largest group contains roughly one-third of the articles in the dataset and is regarded as the current core SNS literature. It contains articles elaborating on SNS themes that have already been around for a while. As mentioned earlier, we look for two signs of recent themes: a coherent subnetwork of proper size and growth in the number of publications. These remaining eight groups not only meet the first condition (not too large, not too small) but also the second one. As exhibited in Fig 1, publications within each of these groups are growing. We thus emphasize our examination on these eight groups. Fig 1 summarizes the characteristics of these eight groups. The information for the largest group is put in the left-most column for reference.

The yearly article growth rate for a group is calculated as the number of articles published in a year divided by the total number of articles accumulated up to the previous year. We define absolute growth rate (AGR) as the three-year average of the 2013, 2014, and 2015 yearly article growth rate. The relative growth rate (RGR) for a group is its AGR compared against the whole dataset’s AGR, which is 0.49. A RGR value higher than 1 indicate that articles in that group grew faster than the overall average. All eight groups have positive AGR while five grow faster than the overall average (RGR greater than 1). The last row in Fig 1 presents the paper growth diagram for each group. All of them show rapid growth in the last few years.

The third row of Fig 1 presents the results of the keyword analysis, which hints at the research focus of each group. The analysis herein counts, in the title and abstract of the articles in that group, the appearance of each term from a keyword list we developed. Each term is counted only once for each paper, even though it is mentioned multiple times. Keywords within each group are listed in the order of their appearance frequency, which is defined as the number of appearances divided by the number of papers in the group. As one can see from the table, Facebook and Twitter stand out on virtually all themes. In fact, with only one exception, one can clearly identify the main social networking website for each group.

For the largest group, the core SNS literature, keywords generally contain studies on young persons’ behavior on SNSs (mostly Facebook), including self-expression, personality, privacy, etc. We thus name the group as “General SNSs”. The themes for the other eight groups are determined without much struggle based on keywords and reading the articles in that group. Their themes are identified as “*Microblogging Health*” (MH), “*Microblogging Politics*” (MP), “*Romantic Relations*” (RR), “*Sentiment and Trend in Microblogs*” (STM), “*Public Relations*” (PR), “*Improving Health*” (IH), “*Journalism*” (JL), and “*Adolescent Health*” (AH).

**Fig 2:**
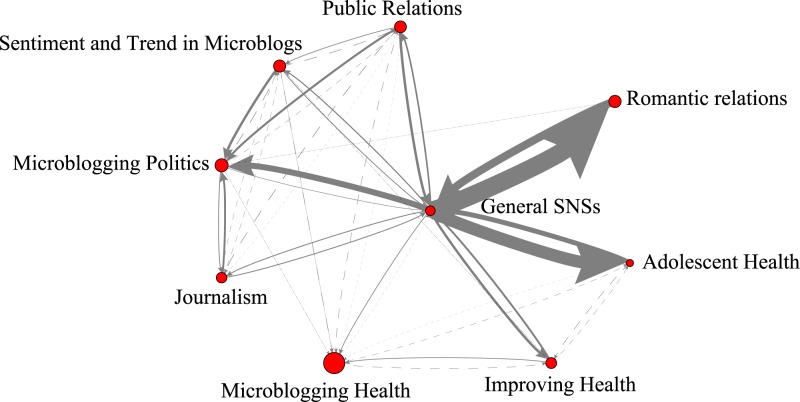
Citation relationships among the SNS core literature (*General SNSs*) and the eight recent research themes. The thickness of each arc is proportional to the number of accumulated citations. Those arcs having less than five citations exhibit dashed lines, where the dash separation is bigger for arcs with a higher number of citations. The sizes of the nodes are drawn proportional to the growth rate of research papers on the theme they represent.

Each group, although identified as being coherent within, is not completely disconnected from the others. Fig 2 presents connections among the eight groups and the core literature. In the figure, each node represents a theme, and the arcs designate the accumulated citation relationships among the articles in each theme. The arcs in the figure indicate knowledge flow with the arrow pointing from the cited themes to the citing themes. The thickness of each arc is proportional to the number of accumulated citations. Those arcs having less than five citations exhibit dashed lines, where the dash separation is bigger for arcs with a higher number of citations.

Four observations are worth noting on the knowledge flow structure and the trend of the SNS research. First, three recent themes—romantic relations, adolescent health, and microblogging politics—are strongly rooted in the core SNS literature and inherit knowledge created in this core literature. Citations from these themes to the core SNS literature are significantly higher than those in the reverse direction. Statistical test results show that the standard score z for GS-RR over RR-GS is 5.469, and those for GS-AH over AH-GS and GS-MP over MP-GS are respectively 4.918 and 5.858, which are all significant at the 0.001 level. Please refer to Table A in S1 Appendix for details.

Second, the theme of politics integrates knowledge from sentiment, journalism, and public relations, probably because they share the same concerns on issues related to mass communication. Table B in S1 Appendix lists the evidence, whereby the standard score z for citations STM-MP, JL-MP, and PR-MP over the case when citations are distributed randomly are all significant. Third, the three themes about health are not connected very much. Aside from addressing different health issues, their knowledge seems to follow from articles in different disciplines.

Fourth and finally, there is a shifting trend of research focus in the related literature from Facebook to Twitter, as there are more recent themes focusing on Twitter than on Facebook, and as the RGRs for four out of five Twitter themes are greater than one. However, that does not mean that research on Facebook is in a complete downward spiral. The literature on Facebook continues to grow, but at a slower rate. The following sections briefly discuss the eight recent research themes.

### 4.1 Microblogging Health

The fastest growing theme is *Microblogging Health*, which began only recently (2012), yet research on the theme surged soon after. This theme contains 35 articles and has grown strongly versus the overall growth rate (RGR = 3.48). Individuals and organizations use microblogging services, typically Twitter, to exchange health-related information, including drug abuse [33–36], physical activity [37], smoking [38, 39], and more recently, e-cigarette [40–43]. These exchanges provide a huge amount of data for researchers to monitor and examine Twitter users’ health behaviors as well as to study how health information is promoted and marketed on Twitter. The results of these studies largely improve our understanding of how Twitter is used in regards to health issues. For example, analyses on e-cigarette-related tweets show that more that 90% of them are commercials [40, 42]. Other interesting studies in this group include a survey of location information available in Twitter [44], and an examination on how Twitter was used in gene patent debate [45]. The huge interest on this theme is exemplified by the fact there is already a review article on it [12], which is a good source for scholars who are interested in further information on the theme.

### 4.2 Microblogging Politics

SNSs, in particular Twitter, provide a good venue for electronic word-of-mouth [46]. Politicians have quickly spotted that and have used SNSs to achieve their own political purposes. The results of their activities provide rich material awaiting analysis. Researchers, in particular in the area of communication and political science, are second to none in catching this new and wide-open research opportunity.

Studies in this category generally attempt to find out how and in what manner politicians use Twitter (and Facebook, in fewer papers) and for what purpose. Empirical data used for these studies are spread across countries in all continents, including the U.S. [47], Canada [48], United Kingdom [49], Norway [50–52], Sweden [52, 53], Austria [54], Turkey [55], South Korea [56], Australia [57], Kuwait [58], etc. The findings consistently show that politicians use these services mostly during an election campaign for the purpose of delivering political messages and self-promotion [47]. Other usages such as interacting with voters and maintaining social relations are also reported [49].

Aside from the how and what questions, an interesting study in this category uses Twitter to predict opinion leaders on Twitter [59]. Twitter users with higher connectivity and issue involvement are found to be better at influencing information flow on Twitter. The study demonstrates that other than answering the how and what questions, one is able to gain more insight analyzing contents that address political issues in SNSs.

### 4.3 Sentiment and Trend in Microblogs

Sentiment analysis, or opinion mining, is the computational treatment of opinion, sentiment, and subjectivity in text [60]. Advancements in computational natural language processing and the wide availability of online data have made sentiment analysis a useful tool in detecting feelings and opinions of any online target group such as customers or voters. Earlier applications of sentiment analysis include polarity classification, temporal prediction of events, political opinion mining, etc.^**54**^ The same technology and data are also be used to detect trends, i.e., the interests and attention of a specific group of users.

Studies in this group include two streams. One focuses on sentiment, while the other emphasizes on trend. Most of the articles in the 1^**st**^ stream analyze sentiment on brands [46, 61, 62] and events[63]. These studies result in some interesting conclusions. For example, popular events, the Oscars for example, are normally associated with increases in negative sentiment strength [63].

News, ongoing events, memes, and commemoratives can trigger trends [64]. Researchers in the 2^**nd**^ stream put their efforts at characterizing trends [65], comparing trends in various countries [66], etc.

### 4.4 Romantic Relations

SNSs have doubtlessly affected many people’s romantic life. Romantic partners can interact and monitor each other on SNSs before establishing, during, and after breaking up the relationship. All these phenomena attract the attention of scholars in communication and psychology fields. Moreove, it took only a few years for this romantic-relations-on-SNSs subject to take a seat in the literature.

Romantic partners examining each other’s profiles and activities on SNSs is one particular phenomenon under the subject of interpersonal electronic surveillance [67], Studies on this group address issues such as will SNS usage induce jealousy or increase happiness [68], if surveillance in a romantic relationship helps breakup recovery [69], the association of attachment style and surveillance [70, 71], etc. Studies in these theme groups also discuss relational intrusion [72, 73] as well as cheating, breakup, and divorce caused by SNSs [74, 75].

### 4.5 Public Relations

Around 43% of articles (22 out of 51) in this group are published in the journals *Public Relations Review* and *Journal of Public Relations Research*, highlighting the public relations tone for this group. The emphasis, nevertheless, is on non-profit and non-governmental organizations’ use of Twitter and Facebook for the purpose of communicating with their stakeholders.

Earlier works [76] study how non-profit organizations use SNSs to advance their organization’s mission and programs and advocate studying Twitter usage in public relations [77]. Subsequent studies focus on public relations for various type of non-profit organization from various aspects [78–81]. While most organizations use SNSs as a one-way communication channel, one study suggests that higher levels of interactivity in an organization may lead to a better relationship [82].

### 4.6 Improving Health

The Internet has certainly opened up new channels for improving one’s personal health other than through visiting a hospital or clinic. A “cyber-visit” through an online website to consult doctors for health caring has become a reality not very long ago [83]. Indeed, SNSs like Facebook have gradually turned into platforms for many disease-specific groups where patients share personal experiences, exchange information related to the specific disease, and receive emotional support. The quality and content of information provided in these groups has become the subject of research. Disease-specific groups dedicated to diabetes seem to be examined most extensively [84–86]. whereas breast cancer [86, 87], colorectal cancer [86], concussions [88], etc. also draw some attention.

SNSs provide an extrinsic method of measuring hospital quality. Scholars find that traditional hospital quality measures are positively correlated with likes to a hospital’s Facebook pages [89] as well as user ratings on SNSs [90]. Some research studies go even further to examine whether Facebook likes can be used to predict health outcomes such as mortality and diseases [91].

### 4.7 Journalism

Journalism is one profession that has been extremely affected by SNS services. Ironically, journalism itself promotes the usage and diffusion of these services through press coverages, be them negative or positive [92]. With the success of Twitter, journalists and the traditional media all are forced to find ways to fit into this new form of communication. This category saw studies that address two major research questions. How are journalists using Twitter? How are media and alike coping with Twitter?

Journalists who do microblogging are also likely to use Twitter for the purpose of establishing ‘personal brands’ [93]. Researchers study questions like if journalists who are Twitter users behave under the journalistic norm [94], how journalists of different genders differ in their use of Twitter [95], whether Twitter expands the range of news sources [96], does news sourcing on Twitter exhibit a gender bias [97], etc. How media as an institution is adopting Twitter is also a subject of study. Furthermore, scholars explore the ways television stations [98] and talk radio [99] use Twitter.

Microblogging has turned into a new kind of citizen journalism platform. It is commonly used by social movement activists to disseminate news in ‘crisis’ situations [100]. Studies in this area find that online activism actually translates into offline activism [101], in which calling for participation is not more predominant than communicating information [102], etc.

### 4.8 Adolescent Health

Many adolescent users of SNSs display messages and pictures related to sex, alcohol, drugs, or violence in their user profile. These behaviors raise questions as to the prevalence of such behaviors and the effects thereof.

The majority of the research in this area observes and analyzes college students’ health-related behavior on Facebook [103–106], while some earlier studies did so on MySpace [107–109]. There are also studies that investigate students’ view on displaying such references through the technique of focus groups [110, 111]. The results of research in this theme generally show that displaying such risky references is quite common among adolescent SNS users, and that such behaviors increase the health risk of their peers.

## 5. Discussions

The recent themes presented above span a wide spectrum of disciplines that are mostly within the areas of social science and healthcare science. This is because that our primary goal is to study SNS’ societal implications rather than their algorithmic aspects and structural characteristics. SNS literature in computer science, physics, mathematics, etc. are excluded in the data collection stage. The themes captured are thus quite reasonable, but readers are reminded to interpret the results with some caution. First, the analysis does not include all the articles we intend to study. Notably missing are several highly recognized papers that are presented in conference [112–114], book chapters [115, 116], and published in journals not listed in WOS [117]. These articles discuss legitimate issues, such as why people use Twitter [112], the closure process on Twitter [113], finding similar users in Facebook [116], etc.

Second, this study is a citation-based analysis. A theme can only be observed if citations among articles addressing similar topics are woven into a recognizable scale. In reality, articles addressing similar topics may not know of other papers’ existence, especially when they are written by scholars in different research areas. Such a fragmented situation reduces the chances of theme formation and the visibility of these articles. For example, we observe that a series of studies on Facebook by a group of complex networks scholars [115–119] are not well noted in the social science literature, and these studies also only barely reference the social science literature. As a result, these articles do not receive proper recognition in the analysis. The same circumstance applies to many articles in our dataset that are published in multidisciplinary or interdisciplinary journals, such as PLOS ONE, Advances in Complex Systems, Complexity, Scientific Report, etc.

Third, the citation network is naively transformed to an undirected network before conducting the cluster analysis. Ideally, the directionality in a citation network should be preserved in the analysis. Fourth, although citation analysis is widely accepted in the research community, it also draws some criticisms, including no discrimination on the level of citation relevancy, unrelated self-citations, etc. Finally, citations change as new articles come onto the scene. Considering that SNS research is a fast developing area, the current result is only a snapshot of this research area at the time we collected the data, which is January 30, 2016.

## 6. Conclusions

This study uncovers the most recent research activities that have congregated into small coherent body of literature. Hopefully, the results presented herein are able to inspire researchers across social science fields to further drive these themes and to enlighten scholars who are not already on the SNS bandwagon with some new directions.

Internet technology has changed the way we interact with others and deal with the world. SNSs are more recent applications of the technology, yet their impacts on everyday life are more dramatic than those earlier applications. Research on the phenomena and issues that involve SNSs is rapidly expanding and still sees no end in sight. A simple count in our dataset on the number of articles published in the period from 2010 to 2015 is 114, 203, 259, 425, 516, and 601, respectively, indicating that SNS research is accelerating at around 100 articles per year since 2010. In addition to the eight recent themes, the direction for further research opportunities is wide open, but where might it lead?

The characteristic of each SNS attracts a unique group of audiences or followers and yields special effects to them. One can thus see discussions regarding self-expression, privacy, and romantic relations typically on Facebook; studies on journalism, politics, and sentiment mostly on Twitter; and explorations of human resource management issues on LinkedIn. Academic works that elaborate on newer yet market-attractive services such as Instagram and Pinterest have begun to appear recently. Pinterest is similar to Twitter in that they both adopt a following and follower interaction model, and users of both can repost (retweet or repin) other users’ contributions [120]. Instagram, in contrast to Facebook that provides general social networking functions, emphasizes on photo and video sharing capabilities [121]. One should expect more research on the social and psychological effects of these new SNSs in the near future.

As mentioned earlier, the majority of SNS articles are currently published in psychology, communication, information & library science, health care, sociology, as well as some interdisciplinary journals, suggesting that current discussions surround the fields of psychology, communication, and health. Other disciplines in social science, for example, law, management, and tourism, receive some attention, but much more issues in these fields can be explored in the future.

Finally, just like Internet, SNSs are global phenomena, being widely accepted across geographical areas and across cultures. Nevertheless, different cultures view and apply them in various ways. Qzone, Facebook’s largest counterpart in China, has around 448 million users (65.1% of the country’s 688 million Internet users) in December 2015 according to a report issued by China Internet Network Information Center [122]. India, the number two country in the world by population, also has a large base of SNS users. The SNS phenomena in these two large markets have not been adequately addressed in the literature. Comparative studies will help us understand local variations of the global phenomena. One recent study, which compares the adoption of SNSs in the United States, China, and India [123], is an example of research moving towards this direction.

## Supporting Information

S1 Appendix Statistical Test Results. (PDF)
