# Supplementary material for: Recent Themes in Social Networking Service Research
Source: PLoS One. 2017 Jan 20;12(1):e0170293. doi: 10.1371/journal.pone.0170293 (PMC5249203; doi:10.1371/journal.pone.0170293)
Supplement: S1 Appendix — (PDF) [file pone.0170293.s001.pdf]

Supporting Information

S1 Appendix. Statistical Test Results

This appendix presents the statistical test results regarding the significance of citation relationships among the research themes depicted in Figure 2. In the figure, the thickness of the line is proportional to the number of citation counts. Whether one relationship is stronger than the other, however, cannot be intuitively determined just by comparing citation counts, because the number of research papers in each theme varies. Themes containing more papers are more likely to induce a high number of citations if those citations are randomly distributed. We adopt statistical tests to examine the significance of citation relationships and calculate the standard score z by applying the following formula:

$$z = \frac{p_1 - p_2}{\sqrt{\frac{p_1 q_1}{n_1} + \frac{p_2 q_2}{n_2}}}, \text{ and } q_1 = 1 - p_1, q_2 = 1 - p_2.$$

Here, p_1 and p_2 are the two citation frequencies to be compared, which are citation counts normalized against the multiple of the number of research papers on each side of a link, n_1 and n_2, respectively.

The 1st test examines the differences between the citations from and to the General SNSs literature for each of the 8 themes. Table A lists the test results, in which the notation GS-RR specifies the citation from RR to GS. The table indicates that citations from three themes - romantic relations (RR), adolescent health (AH), and microblogging politics (MP) - to the General SNSs (GS) literature are higher and statistically significant than those in the reverse direction. For example, the standard score z for GS-RR over RR-GS is 5.469, while those for GS-AH over AH-GS and GS-MP over MP-GS are respectively 4.918 and 5.858, which are all significant at the 0.001 level.

The 2nd test is implemented to check citation differences of each theme pair against the expected case when citations are distributed randomly. In this test, $n_1 = n_2$. Table B shows the results and only lists the links with an actual citation count greater or equal than 5. The results imply that GS-RR, GS-AH, RR-GS, STM-MP, JL-MP, and PR-MP are significantly higher than the case when citations are distributed randomly, while GS-PR, GS-JL, JL-GS, GS-MP, GS-MH, GS-STM, and STM-GS are significantly lower.

Table A. The standard score z among citation pairs (all citations are from other areas to General SNSs and from General SNSs to other areas)

	GS-RR	GS-AH	GS-IH	GS-MP	GS-JL	GS-STM	GS-PR	GS-MH	RR-GS	AH-GS	IH-GS	MP-GS	JL-GS	STM-GS	PR-GS	MH-GS
GS-RR		4.009^{***}	9.767^{***}	10.580^{***}	10.966^{***}	10.978^{***}	10.814^{***}	11.086^{***}	5.469^{***}	8.198^{***}	10.168^{***}	11.556^{***}	10.966^{***}	11.145^{***}	10.339^{***}	11.537^{***}
GS-AH			6.970^{***}	8.114^{***}	8.647^{***}	8.667^{***}	8.433^{***}	8.820^{***}	1.731	4.918^{***}	7.517^{***}	9.519^{***}	8.647^{***}	8.909^{***}	7.760^{***}	9.487^{***}
GS-IH				1.174	2.358^{*}	2.371^{*}	1.947	2.665^{**}	-5.031^{***}	-2.384^{*}	0.707	3.947^{***}	2.358^{*}	2.791^{**}	0.876	3.889^{***}
GS-MP					1.982^{*}	2.020^{*}	1.328	2.501^{*}	-6.094^{***}	-3.715^{***}	-0.345	5.858^{***}	1.982	2.790^{**}	-0.237	5.399^{***}
GS-J						-0.025	-0.581	0.393	-6.680^{***}	-4.571^{***}	-1.688	2.134^{*}	0.000	0.491	-1.770	2.050^{*}
GS-STM							-0.572	0.430	-6.696^{***}	-4.593^{***}	-1.695	2.281^{*}	0.025	0.535	-1.784	2.179^{*}
GS-PR								0.991	-6.455^{***}	-4.249^{***}	-1.237	2.933^{**}	0.581	1.127	-1.258	2.806^{**}
GS-MH									-6.856^{***}	-4.823^{***}	-2.017^{*}	1.701	-0.393	0.077	-2.153^{*}	1.633
RR-GS										3.019^{***}	5.568^{***}	7.546^{***}	6.680^{***}	6.937^{***}	5.781^{***}	7.518^{***}
AH-GS											3.066^{***}	5.865^{***}	4.571^{***}	4.944^{***}	3.315^{***}	5.816^{***}
IH-GS												3.407^{***}	1.688	2.142^{*}	0.121	3.344^{***}
MP-GS													-2.134^{*}	-1.823	-3.933^{***}	0.075
J-GS														0.491	-1.770	2.050^{*}
STM-GS															-2.316^{*}	1.723
PR-GS																3.818^{***}
MH-GS																

Note: *, **, and *** indicate significance level at 0.05, 0.01, and 0.001, respectively. The notation GS-RR specifies the citation from RR to GS. Each cell presents the standard score z for the difference of the link on the left-most column over that on the top row. For example, the standard score z of GS-RR over GS-MP is 10.580^{***}, which means that GS-RR is greater than GS-MP and that this result is statistically significant at the level 0.001.

Table B. The standard score z of the statistical test between actual links and the links that are randomly connected

	GS-RR	GS-AH	RR-GS	STM-MP	JL-MP	PR-MP	AH-GS	IH-MH	PR-STM	MP-JL
Standard score z	9.2174^{***}	6.3623^{***}	4.3376^{***}	2.7427^{**}	2.2589^{*}	2.2205^{*}	1.6351	1.5890	1.1650	0.5171
	GS-IH	IH-GS	PR-GS	GS-PR	GS-JL	JL-GS	GS-MP	GS-MH	GS-STM	STM-GS
Standard score z	-0.7724	-1.4715	-1.8817	-3.0740^{**}	-3.1643^{**}	-3.1643^{**}	-3.2997^{***}	-3.3127^{***}	-3.3391^{***}	-3.7788^{***}

Note: Each cell presents the standard score z of the difference between the specified link and the case when the citations are distributed randomly.
